# Pharmacologic pitfalls in heart failure: A guide to drugs that may cause or exacerbate heart failure. A European Journal of Heart Failure expert consensus document

**DOI:** 10.1002/ejhf.70087

**Published:** 2025-12-11

**Authors:** Amr Abdin, Johann Bauersachs, Magdy Abdelhamid, Suleman Aktaa, Hussam Al Ghorani, Antonio Bayes‐Genis, Jan Biegus, Michael Böhm, Javed Butler, Nicolas Girerd, Marco Metra, Wilfried Mullens, Hadi Skouri, Muthiah Vaduganathan, Seif El Hadidi, Giuseppe M.C. Rosano, Gianluigi Savarese

**Affiliations:** ^1^ Internal Medicine Clinic III, Cardiology, Angiology and Intensive Care Medicine Saarland University Hospital Homburg/Saar Germany; ^2^ Department of Cardiology and Angiology, Hannover Medical School Hannover Germany; ^3^ Faculty of Medicine, Kasr Al Ainy, Cardiology Department Cairo University Giza Egypt; ^4^ Department of Cardiology, St. Paul's Hospital Vancouver BC Canada; ^5^ University Hospital Germans Trias and Pujol Badalona Spain; ^6^ Department of Cardiology, Clinical Department of Intensive Cardiac Care, Wroclaw Medical University, Faculty of Medicine Institute of Heart Diseases Wroclaw Poland; ^7^ Baylor Scott and White Research Institute Dallas TX USA; ^8^ Department of Medicine University of Mississippi Jackson MS USA; ^9^ INSERM, CHU de Nancy Université de Lorraine, and the CardioRenal Integration Clinical Investigation Center Vandoeuvre‐les‐Nancy France; ^10^ Cardiology, ASST Spedali Civili and Department of Experimental and Applied Medicine, Institute of Cardiology University of Brescia Brescia Italy; ^11^ Department of Cardiology, Ziekenhuis Oost‐Limburg Genk Belgium; ^12^ Biomedical Research Institute, Faculty of Medicine and Life Sciences, LCRC UHasselt‐Hasselt University Diepenbeek Belgium; ^13^ Division of Cardiology, Sheikh Shakhbout Medical City Abu Dhabi United Arab Emirates; ^14^ Cardiology Division, Medicine Department, Faculty of Medicine and Medical Sciences Balamand University Balamand Lebanon; ^15^ Cardiovascular Division, Brigham and Women's Hospital, Harvard Medical School Boston MA USA; ^16^ Royal College of Surgeons in Ireland Dublin Ireland; ^17^ National Office of Clinical Audit Dublin Ireland; ^18^ Department of Human Sciences and Promotion of Quality of Life San Raffaele Open University of Rome Rome Italy; ^19^ IRCCS San Raffaele Roma Italy; ^20^ Department of Clinical Science and Education Södersjukhuset; Karolinska Institutet Stockholm Sweden

**Keywords:** Drugs, Harmful, Heart failure, Interaction, Management

## Abstract

Heart failure (HF) exerts a global health burden, often complicated by polypharmacy due to the frequent coexistence of cardiovascular and non‐cardiovascular comorbidities. While guideline‐directed medical therapy and devices have significantly improved outcomes, a range of commonly prescribed medications may inadvertently worsen HF or precipitate decompensation. This expert consensus statement provides a comprehensive overview of drugs known to cause or exacerbate HF, offering practical guidance for clinicians to identify and avoid harmful pharmacologic exposures in this vulnerable population. The review examines the pathophysiological mechanisms, clinical evidence, and guideline‐based recommendations for several drug classes, including antidiabetic agents (e.g. thiazolidinediones, dipeptidyl peptidase‐4 inhibitors), antiarrhythmics (particularly Class I and III), calcium channel blockers, non‐steroidal anti‐inflammatory drugs, antifungals (e.g. itraconazole, amphotericin B), macrolide antibiotics, antihypertensives (e.g. α_1_‐blockers, centrally acting sympatholytics), neurological and psychiatric medications (e.g. carbamazepine, pregabalin, lithium), and selected anaesthetic and anticancer agents such as anthracyclines and vascular endothelial growth factor inhibitors. Each section addresses clinical scenarios where these medications may be contraindicated or require close monitoring. Importantly, this document emphasizes the need for individualized therapy, close review of medication regimens, and collaborative care to minimize iatrogenic harm. The goal is to empower clinicians, pharmacists and nurses to optimize HF treatment while reducing the risk of drug‐induced deterioration. Awareness of these pharmacologic pitfalls is critical to improving clinical outcomes and minimizing preventable adverse events and HF hospitalizations.

## Introduction

Heart failure (HF) is among the fastest‐growing cardiovascular (CV) diseases, posing a significant economic burden on healthcare systems worldwide.^1^, ^2^ Preventing drug–drug interactions and direct myocardial toxicity can potentially reduce hospital admissions, thereby lowering costs and improving patients' quality of life.^3^

**Figure 1:**
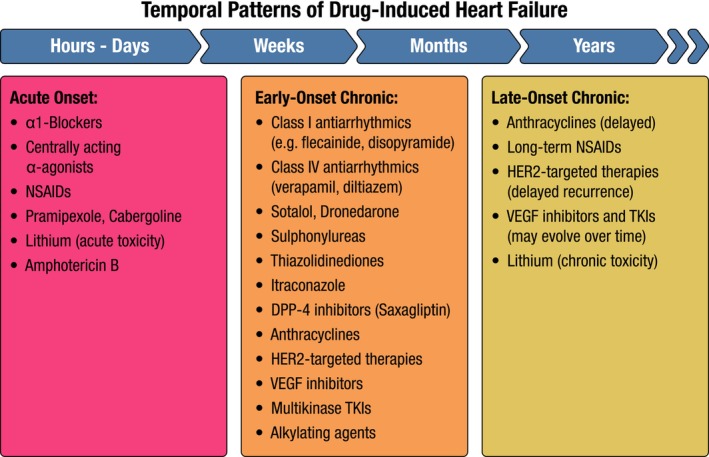
Temporal patterns of drug‐induced heart failure. DPP‐4, dipeptidyl peptidase‐4; HER2, human epidermal growth factor receptor 2; NSAID, non‐steroidal anti‐inflammatory drug; TKI, tyrosine kinase inhibitor; VEGF, vascular endothelial growth factor.

Heart failure is often accompanied by a wide range of CV and non‐CV comorbidities, increasing the number of medications prescribed.^4^, ^5^, ^6^ While necessary, polypharmacy can heighten the risk of adverse drug effects, reduce adherence, and lead to the underprescription or underdosing of guideline‐directed medical therapies (GDMT).^7^, ^8^ Furthermore, certain medications may directly impair cardiac contractility or negatively affect haemodynamics by increasing preload or afterload (*Figure* *1*, *Table* *1*). These effects can precipitate HF in patients without pre‐existing CV diseases or exacerbate HF in those with previously stable conditions^9^ (*Table* *2*).

**Table 1:** Risk profile of drug classes by mechanism of heart failure worsening

Drug class	Fluid retention	Negative inotropy	Arrhythmia	Myocardial toxicity
Antidiabetics				
TZDs	High	Low	Low	Low
DPP4i (saxagliptin)	Moderate	Low	Moderate	Low
Sulfonylureas	Moderate	Low	Low	Low
Antiarrhythmics				
Class Ic	Low	Moderate	High	Low
Sotalol	Low	High	Moderate	Low
Dronedarone	Low	Moderate	High	Low
CCBs	Moderate	High	Low	Low
Antihypertensives				
Doxazosin	High	Low	Low	Low
Moxonidine	Low	High	Low	Low
Minoxidil	Low	Low	High	Low
Anti‐infectives				
Itraconazole	Moderate	High	Low	Low
Amphotericin B	Low	Low	Low	High
Analgesics				
NSAIDs	High	Low	Low	Low
Neurological and psychiatric				
Pramipexole	Low	Low	High	Low
Cabergoline	Low	Low	High	Low
Lithium	Moderate	Low	Moderate	Low
Anticancer				
Anthracyclines	Low	Low	High	High
Alkylating agents	Low	Low	Low	High
Antimetabolites	Low	Low	Low	High
HER2‐targeted therapies	Low	Low	Moderate	High
VEGF inhibitors and multikinase TKIs	Low	Low	Moderate	High
Taxanes	Low	Low	Low	High

CCB, calcium channel blocker; DPP4i, dipeptidyl peptidase‐4 inhibitor; HER2, human epidermal growth factor receptor 2; NSAID, non‐steroidal anti‐inflammatory drug; TKI, tyrosine kinase inhibitor; TZD, thiazolidinedione; VEGF, vascular endothelial growth factor.

**Table 2:** Summary of high‐risk drug classes in heart failure

Drug class	Common agents	Recommendation	Rationale
Analgesic and anti‐inflammatory			
NSAIDs	Ibuprofen, diclofenac	❌ Avoid	Fluid/sodium retention, ↑ BP, renal risk
Corticosteroids	Prednisolone, dexamethasone	❌ Limit/Caution	Fluid/sodium retention, ↑ BP
Antidiabetic			
TZDs	Pioglitazone, rosiglitazone	❌ Avoid	Cause/exacerbate fluid overload
DPP4i (selected)	Saxagliptin, alogliptin	❌ Avoid	↑ HF hospitalization
Sulfonylureas	Glimepiride, glibenclamide	❌ Limit/Caution	Use with caution and close monitoring
Antiarrhythmics			
Non‐DHP CCBs	Verapamil, diltiazem	❌ Avoid in ↓ EF	Negative inotropy
Class Ic	Flecainide, disopyramide	❌ Avoid in structural HD	Proarrhythmic, negative inotropy
Class III	Dronedarone, sotalol	❌ Avoid in HFrEF	Proarrhythmic, negative inotropy
Antihypertensives			
Alpha_1_‐blockers	Doxazosin and prazosin	❌ Avoid	Fluid/sodium retention
Centrally acting α‐adrenergic agonists	Moxonidine	❌ Avoid in HFrEF	Negative inotropy
Anti‐infectives			
Antifungal	Itraconazole, amphotericin B	❌ Avoid	Negative inotropy, myocardial toxicity
Macrolide	Clarithromycin, erythromycin	❌ Limit/Caution especially during COPD treatment	Fluid/sodium retention
Neurological and psychiatric			
Neurological and psychiatric	Pramipexole, cabergoline, lithium	❌ AVOID in ↓ EF	Cause/exacerbate fluid overload Proarrhythmic
Anticancer			
Alkylating agents	Cyclophosphamide, ifosfamide, mitomycin c	❌ Limit/Caution especially in ↓ EF	Risk factors include older age, prior radiation, and cumulative dosing
Anthracyclines	Doxorubicin, epirubicin	❌ Limit/Caution especially in ↓ EF	Dose‐dependent cardiomyopathy
HER2‐targeted therapies	Trastuzumab, pertuzumab, lapatinib	❌ Limit/Caution especially in ↓ EF	Risk factors include prior anthracycline exposure, low baseline LVEF, or comorbidities
VEGF inhibitors and multikinase TKIs	Bevacizumab, sunitinib	❌ Limit/Caution especially in ↓ EF	Underlying cardiac disease

BP, blood pressure; CCB, calcium channel blocker; COPD, chronic obstructive pulmonary disease; DHP, dihydropyridine; DPP4i, dipeptidyl peptidase‐4 inhibitor; EF, ejection fraction; HD, heart disease; HF, heart failure; HFrEF, heart failure with reduced ejection fraction; HER2, human epidermal growth factor receptor 2; LVEF, left ventricular ejection fraction; NSAID, non‐steroidal anti‐inflammatory drug; TKI, tyrosine kinase inhibitor; TZD, thiazolidinedione; VEGF, vascular endothelial growth factor.

The risk of adverse drug–drug interactions increases dramatically with the number of medications prescribed—from 13% in patients taking two drugs to 82% with seven or more.^10^, ^11^ Many of these interactions are particularly harmful to HF patients. Notable examples include non‐steroidal anti‐inflammatory drugs (NSAIDs), non‐dihydropyridine calcium channel blockers (CCBs), and thiazolidinediones (TZD).^9^, ^12^

To minimize risks, clinicians must avoid inappropriate medications, tailor medication regimens to achieve an optimal risk–benefit balance, and remain vigilant to the potential for medications to induce or worsen HF.^3^, ^13^

This statement aims to provide a clinically relevant guide to prescription medications that may cause myocardial toxicity or exacerbate underlying myocardial dysfunction. It synthesizes evidence on potentially inappropriate prescribing to support healthcare providers in optimizing GDMT while minimizing adverse outcomes. By aligning therapeutic strategies with an individual patient's condition, clinicians can improve HF management and enhance clinical outcomes.

## Antidiabetic medications

### Thiazolidinediones

Thiazolidinediones, including rosiglitazone and pioglitazone, are oral insulin‐sensitizing medications that have been widely used in the treatment of type 2 diabetes mellitus (T2DM).^14^ These agents are effective in reducing blood glucose levels with minimal risk of hypoglycaemia and may provide additional benefits on atherosclerosis. However, their clinical utility has been limited by significant concerns regarding their association with HF and other adverse CV outcomes.^15^, ^16^

Shortly after TZDs were introduced into clinical practice, evidence emerged linking their use to an increased risk of oedema and congestive HF. Peripheral oedema is a common adverse effect of TZD therapy, occurring in 3–5% of patients on monotherapy, and increasing to 7.5–8% when combined with other antidiabetic agents, such as sulfonylureas.^14^ Moreover, data from clinical trials and meta‐analyses have consistently highlighted a higher risk of HF events in patients treated with TZDs.^17^, ^18^, ^19^ The DREAM trial evaluated rosiglitazone in individuals at high risk for developing T2DM. Among 2635 patients randomized to rosiglitazone and 2634 to placebo, those receiving rosiglitazone had a significantly higher incidence of HF, with a hazard ratio (HR) of 7.03 (95% confidence interval [CI] 1.60–30.9, *p* = 0.01).^17^ Similarly, in a 6‐month randomized, double‐blind, multicentre trial, patients with T2DM and New York Heart Association (NYHA) class II–III HF (left ventricular ejection fraction [LVEF] ≤40%) were randomized to receive either pioglitazone or glyburide^20^: pioglitazone led to earlier onset of HF and a higher incidence of the composite endpoint of CV mortality and HF‐related hospitalization or emergency visits (13% vs. 8% for glyburide, *p* = 0.024).

Given these findings, contemporary guidelines emphasize caution with TZDs in patients with HF. According to the European Society of Cardiology (ESC) guidelines on HF^2^ and diabetes,^21^ TZDs significantly increase the risk of HF hospitalization, and as a result, these agents are not recommended in patients with diabetes and symptomatic HF due to their potential to exacerbate fluid retention and worsen HF symptoms.

While TZDs offer effective glycaemic control and some vascular benefits, their role in clinical practice has been greatly diminished due to their adverse effects on HF outcomes. For patients with T2DM and established HF or those at high CV risk, alternative glucose‐lowering therapies with proven CV safety, such as sodium–glucose co‐transporter 2 inhibitors, are preferred.

*Clinical advice: Avoid TZDs (rosiglitazone, pioglitazone) in patients with diabetes and HF, as they increase fluid retention and HF hospitalization risk. Use alternative glucose‐lowering therapies with proven CV safety instead*.

### Dipeptidyl peptidase‐4 inhibitors

Several CV outcomes trials have investigated the safety of dipeptidyl peptidase‐4 inhibitors (DPP4is)—including sitagliptin, saxagliptin, alogliptin, and linagliptin—in patients with T2DM, particularly focusing on HF outcomes.^22^, ^23^, ^24^, ^25^, ^26^ The SAVOR‐TIMI 53 trial, which included 16 492 high‐risk T2DM patients (12.8% with HF), found no difference in CV death, myocardial infarction, or stroke with saxagliptin, but did report a significant increase in HF hospitalization (HR 1.27, 95% CI 1.07–1.51), especially in those with elevated natriuretic peptides, prior HF, or chronic kidney disease.^25^ Similarly, a U.S. claims‐based observational study of 7620 patients showed an increased HF hospitalization risk with sitagliptin (odds ratio [OR] 1.84, 95% CI 1.16–2.92).^27^ A meta‐analysis of 84 trials also indicated an increased overall HF risk with DPP‐4is (OR 1.19, 95% CI 1.03–1.37), though variability was observed among agents: saxagliptin showed a significant association, while sitagliptin and linagliptin had no association.^28^, ^29^ Both the EXAMINE trial (alogliptin) and the TECOS trial (sitagliptin) reported no significant differences in HF hospitalizations versus placebo.

Given these mixed findings, particularly the increased risk seen with saxagliptin, DPP‐4is do not appear to uniformly raise HF risk, but saxagliptin is specifically not recommended in T2DM patients with or at high risk for HF, and post‐marketing vigilance remains essential.^2^, ^21^

*Clinical advice: DPP‐4is are generally safe in T2DM, but saxagliptin is associated with increased HF hospitalization risk and should be avoided in patients with or at high risk for HF. Sitagliptin, linagliptin, and alogliptin appear neutral but require continued post‐marketing vigilance*.

### Sulfonylureas

The impact of sulfonylureas on HF risk remains unclear. While two large retrospective cohort studies involving over 110 000 diabetes patients indicated a 20–60% higher mortality rate and a 20–30% increased HF risk as compared to metformin,^30^, ^31^ several major trials—such as UKPDS, NAVIGATOR, and ADOPT—did not report a heightened risk of HF with sulfonylureas.^32^, ^33^, ^34^

*Clinical advice: Sulfonylureas may be associated with a modestly increased risk of HF and mortality compared with metformin in observational studies, but randomized trial data do not confirm this; thus, they should be used cautiously in patients with T2DM and HF, with preference for agents with proven CV safety*.

## Antiarrhythmic medications

### Class I

Several Class I antiarrhythmic drugs, particularly sodium channel blockers such as disopyramide, flecainide, propafenone, and procainamide, have negative inotropic effects that can impair cardiac contractility and potentially trigger or worsen HF.^35^ This is partly due to their impact on L‐type calcium currents and altered sodium–calcium exchange, which reduces intracellular calcium availability. Disopyramide, in particular, has pronounced myocardial depressant properties and has been shown to precipitate HF shortly after initiation, especially in patients with a prior HF history.^36^ Similarly, flecainide can significantly reduce left ventricular (LV) function in patients with pre‐existing dysfunction and has been associated with increased mortality in the CAST trial, reinforcing its contraindication in HF or structural heart disease.^37^ Propafenone and procainamide share similar negative inotropic effects and have been reported to worsen HF, and their use in patients with LV dysfunction is generally discouraged. In patients with HF with preserved ejection fraction (HFpEF) and no significant structural heart disease or ischaemia, the use of Class I antiarrhythmic drugs (such as flecainide or propafenone) may be considered, as no major safety concerns have been reported in this population.^36^, ^38^

*Clinical advice: Avoid Class I antiarrhythmics (including flecainide, disopyramide, propafenone, and procainamide) in HF with reduced ejection fraction (HFrEF) or structural heart disease due to risk of worsening HF. Use may be considered in selected HFpEF patients without structural abnormalities or ischaemia*.

### Class III

#### Ibutilide and sotalol

Ibutilide, administered intravenously, generally does not cause significant haemodynamic changes in patients with severely reduced LVEF ≤35%. However, HF independently increases the risk of ibutilide‐triggered torsades de pointes, likely due to a pre‐existing prolonged QT interval.^39^

Sotalol, a racemic mixture containing both d‐ and l‐isomers, combines beta‐blocking (via the l‐isomer) and Class III antiarrhythmic effects. While racemic sotalol does not raise post‐myocardial infarction mortality, the pure d‐isomer was linked to increased deaths in a specific trial.^40^ Sotalol can impair cardiac contractility and worsen HF, particularly in patients with severe LV systolic dysfunction (LVSD).^41^ Pre‐marketing data, albeit from older studies, reported worsening HF in approximately 3% of patients without prior HF and up to 10% in those with known HF, with risk increasing alongside baseline severity.^42^ Although these data are from the 1990s, the findings are consistent with the drug's negative inotropic properties and subsequent clinical experience. Accordingly, contemporary international guidelines advise against the use of sotalol in patients with significant LVSD, given the potential to exacerbate HF.^2^, ^43^

*Clinical advice: Use ibutilide with caution in HF patients due to increased risk of torsades de pointes. Avoid sotalol in patients with severe LVSD, in line with guideline recommendations and historical safety data*.

#### Dronedarone

Dronedarone is structurally similar to amiodarone and affects multiple ion channels and adrenergic receptors. While it reduced mortality and hospitalizations in patients with atrial fibrillation in the ATHENA trial, other trials painted a more concerning picture.^44^, ^45^ In the ANDROMEDA trial, dronedarone significantly increased mortality in patients with symptomatic HFrEF, leading to the trial's early termination.^46^ Similarly, the PALLAS trial, which studied patients with permanent atrial fibrillation and additional CV risks, was stopped prematurely due to a rise in CV deaths, strokes, and HF hospitalizations.^47^ Dronedarone may worsen HFrEF, especially in unstable patients or those with pre‐existing LV dysfunction, and should not be used in these groups.^9^ It interacts with drugs like digoxin and beta‐blockers, raising the risk of bradyarrhythmias and necessitating careful dose adjustments and monitoring.^3^, ^9^ Caution is also needed when combining dronedarone with CCBs like verapamil or diltiazem due to mutual increases in drug exposure.

*Clinical advice: Dronedarone is contraindicated in patients with symptomatic HFrEF or unstable LV dysfunction due to increased risk of mortality and HF hospitalization. Use with caution when combined with digoxin, beta‐blockers, or non‐dihydropyridine CCBs due to interaction‐related bradyarrhythmia risk*.

### Class IV

Calcium channel blockers, particularly non‐dihydropyridine types like diltiazem and verapamil, are generally contraindicated in patients with HFrEF due to their negative inotropic effects, which can worsen cardiac function.^3^, ^48^ These drugs reduce calcium influx through L‐type calcium channels, leading to bradycardia, slowed atrioventricular conduction, and reduced myocardial contractility. In post‐myocardial infarction patients with signs of pulmonary congestion or reduced ejection fraction, diltiazem was associated with an increased risk of adverse cardiac events.^49^ Moreover, both verapamil and diltiazem can significantly interact with beta‐blockers and CYP3A4 substrates, heightening the risk of bradycardia, atrioventricular block, and worsening HF—especially when administered intravenously, at high doses, or in the presence of conduction abnormalities.^50^ As such, their use should be limited to closely monitored hospital settings if at all considered.

Dihydropyridine CCBs like nifedipine and amlodipine also carry risks in HF. Although they have vasodilatory effects that can counterbalance their negative inotropy, early studies with nifedipine showed clinical deterioration in patients with HF.^51^ Amlodipine has shown neutral effects on mortality and may increase peripheral and pulmonary oedema, though it may reduce uncontrolled hypertension and chest pain. Among the dihydropyridines, only felodipine and amlodipine have been shown to be relatively safe in patients with HF when used as adjuncts to standard therapy for persistent hypertension or angina.^52^, ^53^ Nonetheless, routine use of CCBs—especially non‐dihydropyridines—should be avoided in HFrEF due to the clear risk of worsening HF and hospitalization.

Although the pivotal studies are older, contemporary guidelines consistently recommend avoiding non‐dihydropyridine CCBs in HFrEF. A potential exception may exist in carefully selected patients with LV dysfunction due to atrial fibrillation and uncontrolled ventricular rate, where short‐term use under close monitoring can be considered to achieve rate control and prevent or treat tachycardia‐induced cardiomyopathy.

*Clinical advice: Avoid non‐dihydropyridine CCBs (verapamil, diltiazem) in HFrEF due to negative inotropic effects and risk of worsening HF. Dihydropyridines like amlodipine or felodipine may be used cautiously for persistent hypertension or angina, but routine CCB use in HFrEF is not recommended*.

## Anti‐infective medications

### Itraconazole

Itraconazole, a commonly used antifungal agent, has been linked to cardiotoxic effects, including hypertension, arrhythmias (e.g. premature ventricular contractions, ventricular fibrillation), and both new‐onset and worsening HF.^54^, ^55^, ^56^ Data from the U.S. Food and Drug Administration (FDA) Adverse Event Reporting System between 1992 and 2001 identified 58 cases of HF in patients receiving itraconazole, with 28 hospitalizations and 13 deaths.^54^ While causality could not be definitively established due to confounding factors like pre‐existing heart disease, itraconazole is believed to exert negative inotropic effects, although the exact mechanism remains unclear.

Reports indicate that HF risk increases with higher doses (≥400 mg daily), and peripheral or pulmonary oedema may occur.^55^ As a result, the FDA advises against using itraconazole in patients with LVSD, existing HF, or those at high risk, except when treating life‐threatening fungal infections with no safer alternative.

Additionally, itraconazole is a potent CYP3A4 inhibitor, which significantly raises blood levels of drugs such as CCBs (e.g. verapamil, diltiazem), statins (e.g. simvastatin, atorvastatin), and eplerenone. These combinations can enhance adverse CV effects, so co‐administration requires close monitoring and dose adjustments, and the use of eplerenone with itraconazole is contraindicated in HF patients.

*Clinical advice: Avoid itraconazole in patients with LVSD, existing HF, or high HF risk due to its negative inotropic effects and association with HF and arrhythmias. Use only if no safer antifungal alternative exists. Monitor closely for drug interactions, especially with CYP3A4 substrates like CCBs, statins, and eplerenone—the latter being contraindicated in HF patients receiving itraconazole*.

### Other antifungal medications

Amphotericin B, an antifungal agent, is associated with infusion‐related adverse reactions such as chest discomfort, shortness of breath, low oxygen levels, rapid heartbeat, and hypotension.^57^ These symptoms typically resolve after stopping the infusion, but severe reactions may necessitate permanent discontinuation. Importantly, overdosing—defined as exceeding 1.5 mg/kg/day—can lead to life‐threatening cardiac or cardiorespiratory arrest, highlighting the need for cautious dosing.^58^, ^59^, ^60^

Multiple reports have documented new‐onset dilated cardiomyopathy and HF in patients receiving both conventional and liposomal forms of amphotericin B.^57^ In all cases, cardiac function improved and HF symptoms resolved within 10 days to 6 months after stopping the drug, suggesting a reversible cardiotoxic effect.

In addition, amphotericin B commonly causes hypokalaemia, which can enhance the toxicity of digoxin, thereby increasing the risk of arrhythmias and worsening HF.^58^, ^59^, ^60^

*Clinical advice: Use amphotericin B with caution due to risk of infusion‐related cardiopulmonary reactions, dose‐dependent cardiotoxicity, and reversible HF. Avoid overdosing (>1.5 mg/kg/day), monitor for hypokalaemia, and adjust digoxin therapy as needed to reduce arrhythmia and HF risk*.

### Other antibiotics (or antimicrobials)

Among antimicrobials, macrolide antibiotics, particularly clarithromycin and erythromycin, have been associated with increased CV risks in patients with respiratory infections and underlying cardiopulmonary disease.^61^, ^62^, ^63^ Use of clarithromycin during acute exacerbations of chronic obstructive pulmonary disease (COPD) or community‐acquired pneumonia (CAP) was linked to a significantly higher incidence of CV events (HR 1.48 and 1.68, respectively).^63^ In COPD cohorts, clarithromycin users exhibited a higher frequency of congestive HF or LVSD (11.4% vs. 5.3%) compared to non‐users. Clarithromycin was also associated with increased CV mortality in COPD exacerbations (HR 1.52), though no significant association was seen with all‐cause mortality or with outcomes in CAP patients.^62^ Erythromycin use during CAP hospitalizations was similarly associated with elevated risks of hospital‐acquired cardiac events (HR 1.68) and HF (HR 2.08). Conversely, azithromycin and clarithromycin showed no significant increase in cardiac events in other contexts (adjusted HRs 0.89 and 1.06, respectively).

Other antimicrobials important to consider in HF include fluoroquinolones, which prolong the QT interval and increase the risk of arrhythmias.^62^ Trimethoprim/sulfamethoxazole raises hyperkalaemia risk, especially in patients on renin–angiotensin system inhibitors or mineralocorticoid receptor antagonists. Intravenous β‐lactam antibiotics like piperacillin–tazobactam may exacerbate fluid overload due to sodium content. Hence, antimicrobial selection in HF patients should carefully balance infection control efficacy with CV safety.^9^

*Clinical advice: In HF patients, avoid macrolides like clarithromycin and erythromycin due to increased risk of HF and CV events, especially during COPD or CAP treatment. Prefer alternatives like azithromycin when appropriate*.

## Antihypertensive drugs

### Alpha_1_‐blockers

Alpha_1_‐blockers such as doxazosin and prazosin induce arterial and venous vasodilatation by inhibiting postsynaptic α_1_‐adrenergic receptors. However, their use in HF is problematic.^64^ In the ALLHAT trial, the doxazosin arm was terminated early due to a doubling of HF risk compared to chlorthalidone.^65^, ^66^ This increased risk was likely due to fluid retention, insufficient blood pressure control, or the unmasking of pre‐existing LVSD, rather than direct causation of HF. Similarly, in the V‐HeFT I trial, prazosin showed no mortality benefit in patients with HFrEF, unlike the combination of hydralazine and isosorbide dinitrate, which significantly reduced mortality.^65^

Regarding uroselective (e.g. tamsulosin) and non‐uroselective (e.g. prazosin, terazosin, doxazosin) α_1_‐blockers used for benign prostatic hyperplasia in patients with HF, evidence remains limited. Most data are extrapolated from earlier studies. A retrospective analysis of 388 HF patients with benign prostatic hyperplasia found no increase in mortality or HF rehospitalization among those receiving both α_1_‐blockers and β‐blockers.^67^ However, in the absence of β‐blocker therapy, α_1_‐blockade was associated with a significant increase in HF hospitalizations. This may be due to unopposed β_1_ stimulation, leading to neurohormonal activation and fluid retention. Moreover, chronic α_1_‐blockade might lead to tachyphylaxis and elevated norepinephrine levels. While the exact mechanism remains unclear, current evidence suggests that α_1_‐blockers—uroselective or not—may worsen outcomes in patients with existing HF.

*Clinical advice: Avoid α*_*1*_ *‐blockers, including uroselective agents, in patients with HF—especially in the absence of β‐blocker therapy—due to increased risk of HF hospitalization and lack of mortality benefit. If required for benign prostatic hyperplasia, use with caution and only in combination with β‐blockers*.

### Centrally acting α‐adrenergic agonists

Heart failure is associated with heightened sympathetic nervous system activity, which correlates strongly with increased mortality. The proven success of β‐blockers in reducing mortality and reversing adverse cardiac remodelling in patients with reduced LVEF sparked interest in other strategies aimed at suppressing sympathetic activation.^68^

Centrally acting α_2_‐adrenergic agonists like clonidine and moxonidine reduce sympathetic nerve outflow, thereby lowering plasma norepinephrine levels and blood pressure. In animal studies, clonidine was shown to improve survival in HF models.^69^ Small clinical trials in humans also demonstrated beneficial haemodynamic effects—such as reduced preload, enhanced stroke volume, and more than 50% reductions in plasma norepinephrine levels with clonidine dosed at 0.15 mg twice daily. However, its use has been linked to adverse effects including bradycardia and atrioventricular conduction abnormalities.^70^, ^71^

Moxonidine, an imidazoline receptor agonist, was studied in a large randomized trial involving patients with NYHA class II–IV HF.^72^, ^73^ The trial was terminated early due to an unexpected increase in mortality among those receiving moxonidine (5.5%) compared with placebo (3.4%), despite reductions in norepinephrine levels and evidence of LV reverse remodelling. It is speculated that the abrupt and substantial reduction in sympathetic tone may have led to harmful myocardial depression by limiting compensatory β‐adrenergic support during acute stress. Therefore, moxonidine should also be avoided in patients with HFrEF due to its detrimental clinical effects despite potential blood pressure‐lowering benefits.

*Clinical advice: Avoid centrally acting α*_*2*_ *‐agonists like moxonidine in HFrEF due to increased mortality risk despite sympathetic suppression. Clonidine may have haemodynamic benefits but use cautiously because of bradycardia and conduction disturbances*.

### Minoxidil

Minoxidil is a potent arterial vasodilator with minimal venous effects. While it has been shown to improve LVEF in small studies—such as a trial where patients treated with minoxidil 20 mg twice daily experienced a significant increase in LVEF from 29.6% to 42.7% over 3 months—it does not lead to improvements in symptoms or exercise capacity.^74^ More importantly, its use in HF is associated with a higher incidence of adverse outcomes, including increased diuretic requirements, angina, arrhythmias, worsening HF, and mortality. Due to these safety concerns, minoxidil is not recommended in patients with HF.^75^

*Clinical advice: Minoxidil is not recommended for HF patients due to increased risks of worsening HF, arrhythmias, angina, and mortality, despite some improvement in LVEF*.

## Analgesics

### Non‐steroidal anti‐inflammatory drugs

Non‐steroidal anti‐inflammatory drugs remain among the most frequently used medications globally, with both prescription and over‐the‐counter formulations widely available.^3^, ^76^ Their mechanism of action primarily involves inhibition of cyclooxygenase (COX) enzymes, which reduces prostaglandin synthesis.^77^ Traditional NSAIDs (e.g. ibuprofen, diclofenac, indomethacin) non‐selectively inhibit both COX‐1 and COX‐2, whereas COX‐2 inhibitors (coxibs, such as celecoxib) selectively target COX‐2, which is up‐regulated during inflammation. While COX‐2 selectivity reduces gastrointestinal adverse effects, the inhibition of COX‐1 by non‐selective NSAIDs impairs platelet function, compromises gastric mucosal protection, and disrupts renal autoregulation.^3^

In patients with or at risk for HF, NSAIDs—regardless of selectivity—can worsen volume status due to sodium and water retention, blunt the efficacy of diuretics and renin–angiotensin system inhibitors, increase systemic vascular resistance, and potentially precipitate or exacerbate HF.^78^, ^79^ Large‐scale observational studies, such as the Rotterdam Study and Danish national registries, have consistently linked NSAID use with increased risks of new‐onset and recurrent HF, especially in elderly individuals, patients with comorbidities (e.g. diabetes, renal dysfunction, hypertension), and those on concurrent diuretic therapy.^80^, ^81^ Among elderly HF patients, NSAID use has been linked with up to a twofold increase in HF hospitalization, especially when used concurrently with diuretics.^77^, ^80^ Notably, agents with a longer half‐life appear to confer greater risk than those with shorter durations of action. Patients with established HF who receive NSAIDs have shown markedly increased rates of hospitalization and mortality. For example, filling a single NSAID prescription after an HF diagnosis can raise the risk of HF relapse nearly tenfold.^80^

More recent data continue to confirm the CV risks of NSAIDs. A 2016 large‐scale case–control study using European healthcare databases^82^ found that current use of NSAIDs—including both non‐selective agents and coxibs—was associated with a significantly increased risk of hospital admission for HF. This risk was dose‐dependent and highest for agents like diclofenac, indomethacin, piroxicam, and rofecoxib. Celecoxib, while generally considered safer, still showed increased CV risk at higher doses or in high‐risk patients.^12^

Given this growing body of evidence, the ESC guidelines recommend avoiding NSAIDs and COX‐2 inhibitors in patients with HF, particularly those with HFrEF.^2^ These agents should be used with great caution—if at all—in high‐risk populations. Clinicians are encouraged to consider alternative analgesics and anti‐inflammatory strategies when treating patients with or at risk of HF (*Table* *3*).

**Table 3:** Summary of high‐risk medications and safer alternatives (pocket card)

Drug class	Examples	Safer alternatives	Evidence strength^a^
Analgesic			
NSAIDs/COX‐2 inhibitors	Ibuprofen, diclofenac, celecoxib	Paracetamol, topical NSAIDs	Strong
Antidiabetic			
TZDs	Pioglitazone, rosiglitazone	SGLT2 inhibitors or metformin	Strong
DPP4i (selected)	Saxagliptin	Sitagliptin, linagliptin	Strong
Sulfonylureas	Glimepiride, glibenclamide	SGLT2 inhibitors or metformin	Strong
Antiarrhythmics			
Class Ic (↓ EF)	Class Ic (flecainide, disopyramide)	Amiodarone (with monitoring)	Strong
Class III (↓ EF)	Sotalol, dronedarone	Amiodarone (with monitoring)	Moderate (sotalol), strong (dronedarone)
Non‐DHP CCBs (↓ EF)	Verapamil, diltiazem	Amlodipine, felodipine	Moderate
Antihypertensives			
Alpha_1_‐blockers	Doxazosin, prazosin	Other antihypertensives per HF guidelines	Strong
Centrally acting α‐adrenergic agonists (↓ EF)	Moxonidine, clonidine	Other antihypertensives per HF guidelines	Strong
Anti‐infectives			
Macrolides	Clarithromycin, erythromycin	Azithromycin	Moderate
Antifungals	Itraconazole	Fluconazole	Moderate
Neurological and psychiatric			
Dopamine agonists (↓ EF)	Pramipexole, cabergoline	Ropinirole, pergolide	Moderate
Lithium	Lithium carbonate	Valproate, lamotrigine	Moderate
Anticancer			
Anthracyclines (↓ EF)	Doxorubicin, epirubicin	Liposomal formulations, dexrazoxane	Strong
VEGF inhibitors (↓ EF)	Sunitinib, bevacizumab	No safer alternative – use only if essential with close monitoring and BP control	Moderate
HER2‐targeted therapies (↓ EF)	Trastuzumab, pertuzumab, lapatinib	No safer alternative – use only if essential with close monitoring and BP control	Strong
Alkylating agents (↓ EF)	Cyclophosphamide, ifosfamide, mitomycin c	No safer alternative – use only if essential with close monitoring and BP control	Moderate

BP, blood pressure; CCB, calcium channel blocker; COX‐2, cyclooxygenase‐2; DHP, dihydropyridine; DPP4i, dipeptidyl peptidase‐4 inhibitor; EF, ejection fraction; HER2, human epidermal growth factor receptor 2; HF, heart failure; NSAID, non‐steroidal anti‐inflammatory drug; SGLT2, sodium–glucose co‐transporter 2; TZD, thiazolidinedione; VEGF, vascular endothelial growth factor.

^a^ Strong = supported by randomized controlled trials and/or meta‐analyses; Moderate = supported by observational studies or subgroup analyses; Weak = mainly case reports, pharmacovigilance, or limited data.

*Clinical advice: NSAIDs, including both non‐selective and COX‐2 selective agents, should be avoided or used with extreme caution in patients with HF*.

## Anaesthetics

With the increasing prevalence of HF and ageing populations, a growing number of patients with compromised cardiac function are undergoing surgical procedures. These patients face elevated perioperative risk, including higher morbidity, mortality, and healthcare utilization. For instance, Hammill *et al*.^83^ reported a 63% increase in operative mortality and a 51% increase in 30‐day all‐cause readmission among patients with HF compared to those without HF or coronary artery disease. This heightened vulnerability is partly attributable to the cardiodepressant effects of general anaesthetics, which can worsen underlying cardiac dysfunction.

### Inhalational anaesthetics

Volatile anaesthetics—such as isoflurane, sevoflurane, and desflurane—are commonly used for maintenance of general anaesthesia due to their relatively stable haemodynamic profile and myocardial preconditioning properties. These agents can reduce the required dose of intravenous anaesthetics and have been associated with decreased myocardial ischaemia–reperfusion injury.^3^ However, agents like halothane and enflurane are generally avoided due to their pro‐arrhythmic and cardiodepressant properties.^84^ While inhalational anaesthetics are preferred for maintenance in patients with ventricular dysfunction, their use for induction is limited by airway irritation, slower onset, and haemodynamic instability, especially in patients with reduced LVEF.^85^

*Clinical advice: Volatile anaesthetics like isoflurane, sevoflurane, and desflurane are preferred for maintenance of anaesthesia in patients with ventricular dysfunction due to their myocardial protective effects and stable haemodynamics. However, agents such as halothane and enflurane should be avoided because of their cardiodepressant and pro‐arrhythmic risks*.

### Propofol

Propofol is the most widely used intravenous anaesthetic for both induction and maintenance of anaesthesia.^3^ It acts via potentiation of gamma‐aminobutyric acid receptors, resulting in dose‐dependent vasodilatation and myocardial depression. While the negative inotropic effects are minimal at standard doses, systemic hypotension can occur due to reduced preload and afterload, especially in patients with HF.^86^ Propofol also blunts baroreceptor reflexes, leading to suppressed sympathetic outflow, which may exacerbate hypotension. Nonetheless, it has cardioprotective and antiarrhythmic effects via free radical scavenging and ischaemic preconditioning, making it a viable option in carefully monitored settings.^86^ For total intravenous anaesthesia, it is typically administered alongside opioids and benzodiazepines.

*Clinical advice: Propofol can be used safely in HF patients under careful monitoring*.

### Etomidate

Etomidate is favoured for induction of anaesthesia in haemodynamically unstable patients, as it causes the least CV depression among anaesthetic agents.^87^ It does not trigger histamine release and preserves myocardial contractility. However, its routine use beyond induction is limited by its inhibition of adrenal steroidogenesis, which may impair the stress response during the perioperative period.

*Clinical advice: A single induction dose is generally considered safe, even in patients with advanced HF*.

### Ketamine

Ketamine, a dissociative anaesthetic, exhibits a complex pharmacodynamic profile with both negative inotropic and sympathomimetic properties. The latter typically increases heart rate, blood pressure, and cardiac output through inhibition of catecholamine reuptake. However, in patients with severe LV dysfunction or depleted catecholamine stores, these compensatory mechanisms may fail, leading to haemodynamic instability and worsening cardiac performance.^88^ Additionally, ketamine increases myocardial oxygen consumption, rendering it unsuitable in patients with coronary artery disease, tachyarrhythmias, or hypertension.

*Clinical advice: Ketamine increases heart rate and blood pressure through sympathetic stimulation but can cause instability in severe HF due to depleted catecholamines. It also raises myocardial oxygen demand, so it is unsafe in coronary artery disease, arrhythmias, or hypertension and severe HF*.

### Dexmedetomidine

Dexmedetomidine is a selective α_2_‐adrenergic agonist used for intraoperative sedation and postoperative analgesia. It exerts sympatholytic and vagomimetic effects, potentially causing bradycardia and hypotension, particularly during bolus dosing or in volume‐depleted patients. In small observational studies, it demonstrated relative haemodynamic stability in paediatric HF populations, but caution is warranted. Dexmedetomidine is being increasingly adopted in cardiac anesthesia for its opioid‐sparing properties and minimal respiratory depression, though its safety in advanced HF remains to be fully defined.

*Clinical advice: Use dexmedetomidine cautiously in HF patients due to risks of bradycardia and hypotension; monitor haemodynamics closely, especially during bolus dosing or in volume‐depleted individuals*.

## Neurological and psychiatric medications

### Antiepileptic medications

Antiepileptic drugs, while essential for seizure control, neuropathic pain, and mood stabilization, may adversely impact cardiac function in vulnerable populations. Certain agents have been associated with worsening HF and LVSD, particularly in elderly or CV‐compromised patients.

Carbamazepine is a voltage‐gated sodium channel blocker that preferentially binds to the inactivated state of the channel, delaying its reactivation and reducing neuronal excitability. While widely used as an antiepileptic, mood stabilizer, and treatment for neuropathic pain, carbamazepine has been implicated in cardiotoxicity in overdose settings. Case reports describe the development of severe LVSD (LVEF <35%) in patients following carbamazepine overdose—even in those without underlying cardiac disease.^89^, ^90^, ^91^ Such cases underscore the drug's negative inotropic potential, particularly at toxic serum concentrations. Although uncommon in therapeutic dosing, caution is advised in HF patients with pre‐existing LV dysfunction, as the safety margin is narrow in this population.

Pregabalin is a structural analogue of γ‐aminobutyric acid that binds to the α2δ subunit of voltage‐gated calcium channels in the central nervous system.^92^ It exerts analgesic, anxiolytic, and anticonvulsant effects and is commonly prescribed for neuropathic pain and generalized anxiety. However, post‐marketing surveillance has revealed associations with new‐onset HF or worsening LVSD, especially in elderly patients with pre‐existing CV conditions.^93^ While the exact mechanism remains unclear, pregabalin‐induced calcium channel modulation may impair myocardial calcium handling, contributing to cardiac decompensation in vulnerable individuals.

Moreover, clinical trials have consistently shown that pregabalin increases the incidence of peripheral oedema and weight gain—both of which are clinically relevant in patients with HF. These adverse effects may result from increased capillary permeability or sodium retention, and they have been observed in patients with and without a prior HF diagnosis.^93^ Given these findings, pregabalin should be used with caution in patients with LV dysfunction, especially those with borderline fluid status or at risk of decompensation.

*Clinical advice: Carbamazepine should be avoided in patients with unstable HF or used with caution at therapeutic doses, particularly in the elderly or those on multiple negative inotropes (e.g. CCBs, antiarrhythmics). Pregabalin should be used cautiously in patients with HF, particularly those with HFrEF, and fluid status should be monitored closely. Alternative agents with neutral haemodynamic profiles, such as duloxetine (for neuropathic pain), may be considered where clinically appropriate*.

### Anti‐parkinsonian medications and heart failure risk

Several dopamine agonists used in the management of Parkinson's disease (PD) have been implicated in an increased risk of HF, particularly in older populations and during the early phases of treatment.^94^ Observational data suggest that the risk may vary depending on the specific agent used.

In a large population‐based cohort of 26 814 users of anti‐parkinsonian drugs, current use of any dopamine agonist was associated with a 58% increased risk of HF compared to non‐use (rate ratio [RR] 1.58, 95% CI 1.26–1.96).^95^ The risk was particularly elevated with pramipexole (RR 1.86, 95% CI 1.21–2.85) and cabergoline (RR 2.07, 95% CI 1.39–3.07). Notably, other dopamine agonists such as ropinirole and pergolide were not linked to increased HF risk in this analysis.

Another retrospective cohort study involving 25 459 PD patients found that among non‐ergot dopamine agonists, only pramipexole was significantly associated with a higher risk of HF (OR 1.61, 95% CI 1.09–2.38), particularly within the first 3 months of treatment and in patients aged 80 years or older.^96^ This signal led the U.S. FDA to issue a warning in 2012 about a potential HF risk associated with pramipexole use in PD patients.^97^

Despite these findings, the overall CV safety profile of non‐ergot dopamine agonists appears to be acceptable in the broader population of PD patients. There is no clear association with increased major adverse CV events or all‐cause mortality when compared to levodopa monotherapy. Therefore, careful patient selection and close monitoring—especially in elderly individuals or those with preexisting cardiac disease—are warranted when initiating pramipexole or cabergoline.

*Clinical advice: Use dopamine agonists pramipexole and cabergoline with caution in individuals with existing cardiac disease or advanced age, and closely monitor for signs of HF. Alternative dopamine agonists like ropinirole and pergolide may be safer options in high‐risk patients*.

### Others

Lithium, a widely used mood stabilizer for long‐term management of bipolar disorder, has been associated with cardiotoxic effects despite its established psychiatric efficacy. It alters sodium transport in nerve and muscle cells and affects catecholamine metabolism.^3^ While effective, lithium has a narrow therapeutic index, and even at therapeutic serum levels (0.6–1.2 mEq/L), it can produce serious cardiac adverse events.^98^

Multiple case reports and small series have linked lithium to a range of cardiac conduction abnormalities, including sinus node dysfunction, atrioventricular block, premature ventricular contractions, and bradyarrhythmias. Electrocardiographic changes such as T‐wave depression and more severe conditions like interstitial myocarditis and lithium‐induced cardiomyopathy have also been reported.^98^, ^99^ In some patients, lithium has precipitated new‐onset HF or peripheral oedema, even in the absence of pre‐existing cardiac disease.^100^

The underlying mechanisms of lithium‐induced cardiotoxicity are not fully elucidated but may include myofibrillar degeneration with myocardial lymphocyte infiltration, direct inhibition of calcium influx in cardiac pacemaker cells, and autonomic dysregulation, including adrenergic stimulation.^99^

**Figure 2:**
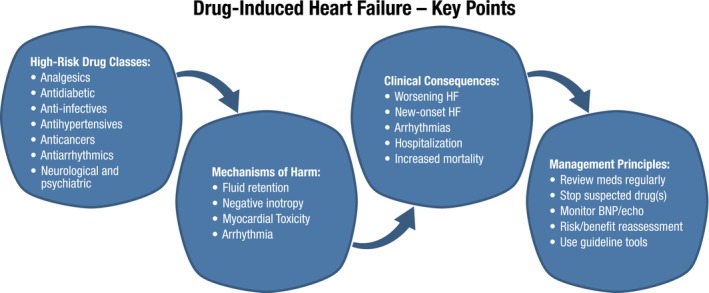
Key points of drug‐induced heart failure (HF) with a summary of drug classes, mechanisms of damage, clinical consequences, and treatment principles. BNP, B‐type natriuretic peptide.

Importantly, most cardiac side effects resolved upon lithium discontinuation, highlighting the importance of early recognition (*Figure* *2*). Given these risks, lithium is contraindicated in patients with HFrEF^2^ (*Table* *4*).

**Table 4:** Summary of high‐risk medications according to heart failure phenotype (pocket card)

Drug class	Avoid in HFrEF	Avoid in HFmrEF	Avoid in HFpEF
Analgesic			
NSAIDs/COX‐2 inhibitors	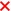 Yes	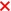 Yes	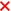 Yes
Antidiabetic			
TZDs (pioglitazone, rosiglitazone)	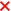 Yes	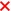 Yes	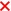 Yes
DPP4i (saxagliptin, alogliptin)	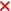 Yes (especially saxagliptin)	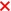 Yes	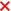 Yes (especially saxagliptin)
Sulfonylureas	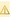 Use with caution	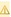 Use with caution	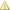 Use with caution
Antiarrhythmics			
Class Ic (flecainide, disopyramide)	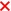 Yes	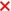 Yes	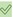 In selected patients
Class III (dronedarone, sotalol)	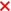 Yes	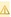 Use with caution	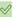 In selected patients
Non‐DHP CCBs	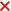 Yes	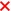 Yes	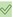 Generally safe
Antihypertensives			
Alpha_1_‐blockers (doxazosin, prazosin)	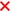 Yes	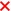 Yes	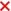 Yes
Moxonidine	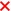 Yes	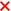 Yes	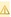 Use with caution
Anti‐infectives			
Macrolides (clarithromycin, erythromycin)	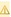 Use with caution	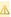 Use with caution	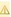 Use with caution
Itraconazole and amphotericin B	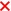 Yes	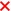 Yes	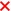 Yes
Neurological and psychiatric			
Pramipexole, cabergoline	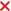 Yes	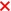 Yes	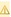 Use with caution
Lithium	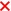 Yes	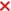 Yes,	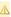 Use with caution
Anticancer			
Anthracyclines	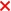 Yes, if essential with close monitoring	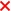 Yes, if essential with close monitoring	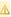 Use with caution
VEGF inhibitors	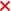 Yes, if essential with close monitoring	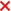 Yes, if essential with close monitoring	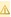 Use with caution
HER2‐targeted therapies	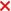 Yes, if essential with close monitoring	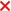 Yes, if essential with close monitoring	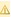 Use with caution
Alkylating agents	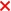 Yes, if essential with close monitoring	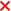 Yes, if essential with close monitoring	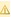 Use with caution

CCB, calcium channel blocker; DPP4i, dipeptidyl peptidase‐4 inhibitor; HFmrEF, heart failure with mildly reduced ejection fraction; HFpEF, heart failure with preserved ejection fraction; HFrEF, heart failure with reduced ejection fraction; HER2, human epidermal growth factor receptor 2; NSAID, non‐steroidal anti‐inflammatory drug; TKI, tyrosine kinase inhibitor; TZD, thiazolidinedione; VEGF, vascular endothelial growth factor.

Safer alternatives for mood stabilization in this population include valproic acid and lamotrigine, both of which lack the cardiotoxicity profile of lithium and are generally considered CV neutral.^3^

Beyond lithium, certain antipsychotics have also been linked to severe cardiotoxicity. Myocarditis and cardiomyopathy, although rare, are recognized and potentially fatal complications of clozapine therapy.^101^, ^102^ Registry data from the Australian Adverse Drug Reaction Unit estimated the incidence of clozapine‐induced myocarditis at 0.7–1.2% over a 10‐year period. These events typically occurred within the first 2 months of treatment, appeared unrelated to dose, and were associated with a recovery rate of 52%, while 10% of affected patients died.^103^ Olanzapine, though less commonly implicated, has also been reported in isolated cases of myocarditis and cardiomyopathy.^104^ Given these risks, clinicians should maintain a high index of suspicion for early cardiac symptoms during initiation of these agents.

*Clinical advice: Lithium is contraindicated in patients with HFrEF. For mood stabilization in patients with HF, safer alternatives like valproic acid or lamotrigine, which have no known cardiotoxicity, are preferred. Caution is warranted with clozapine and, to a lesser extent, olanzapine, due to their potential to cause myocarditis and cardiomyopathy, particularly in the early phase of therapy*.

## Beta_2_‐agonists in chronic obstructive pulmonary disease

Inhaled β_2_‐adrenergic agonists—such as salbutamol, salmeterol, formoterol, and terbutaline—represent a cornerstone in the symptomatic treatment of COPD due to their bronchodilatory effects. However, these agents possess positive chronotropic and inotropic properties, which may exacerbate CV stress and lead to adverse outcomes in patients with underlying HF.^105^

A growing body of evidence from observational studies and large registries has raised concern about the CV safety profile of β_2_‐agonists in this population.^105^, ^106^, ^107^ Use of both short‐ and long‐acting inhaled β_2_‐agonists has been linked to an increased risk of myocardial infarction, sudden cardiac death, and acute decompensated HF. In the CHARM programme, bronchodilator use independently predicted higher rates of HF hospitalization (HR 1.49) and major adverse CV events (HR 1.32).^108^

Further support comes from real‐world registry data, which demonstrated that among more than 160 000 HF hospitalizations, the administration of short‐acting inhaled bronchodilators within the first 48 h was associated with worse in‐hospital outcomes, even in patients without a COPD diagnosis.^108^ Additionally, the use of systemic sympathomimetic drugs following hospital discharge was significantly associated with increased risk of arrhythmia‐related readmission (OR 4.0, 95% CI 1.0–15.1), with systemic formulations posing a greater risk than inhaled alternatives.^109^

Despite these findings, not all studies indicate a detrimental long‐term impact. A retrospective analysis that adjusted for multiple confounders, including B‐type natriuretic peptide levels, found no significant association between β_2_‐agonist use and all‐cause mortality in patients with HF, suggesting that the risk may be context‐dependent and influenced by patient selection and severity of illness.^110^

From a clinical perspective, oral β_2_‐agonists should be strictly avoided in all patients with HF due to their systemic sympathomimetic effects and the high potential for harm. Inhaled β_2_‐agonists, while sometimes necessary, should be used with great caution, and efforts should be made to minimize both the dose and frequency of administration.

In patients requiring frequent rescue therapy, clinicians should consider transitioning to alternative maintenance strategies, such as inhaled corticosteroids and/or long‐acting muscarinic antagonists, which may offer better safety in the HF population.^111^ Moreover, long‐acting β_2_‐agonists have been shown to increase the risk of digoxin‐induced arrhythmias, posing an additional concern in patients treated with digitalis.

*Clinical advice: While β*_*2*_ *‐agonists remain essential for the management of COPD, their use in patients with coexisting HF requires careful risk–benefit evaluation, prudent prescribing, and close monitoring to mitigate potential CV harm*.

## Corticosteroids

Excess glucocorticoid exposure promotes sodium and fluid retention, induces multiple CV risk factors (including obesity, insulin resistance, glucose intolerance, dyslipidaemia, and hypertension), accelerates atherosclerosis, and ultimately increases the risk of developing HF.^112^ Elevated serum cortisol levels have been shown to independently predict mortality in HF patients, with a HR of 2.72 for the highest versus lowest tertile.^113^ High‐dose glucocorticoid therapy has been linked to a higher incidence of CV events and identified as an independent risk factor for HF (OR 2.66, 95% CI 2.46–2.87), particularly in patients with rheumatoid arthritis or COPD.^114^, ^115^ The risk of HF appears dose‐dependent: OR 1.95 for low daily doses (<7.5 mg prednisolone equivalent), OR 2.27 for medium doses (7.5–20 mg), and OR 3.69 for high doses (>20 mg).^115^

Mineralocorticoids, such as fludrocortisone, may counteract the therapeutic benefits of mineralocorticoid receptor antagonists. In Addison's disease, excessive mineralocorticoid replacement has been implicated in the development of LVSD.^116^ Long‐term follow‐up studies report that congestive HF occurred in nearly one‐third of patients with Addison's disease treated with fludrocortisone.^116^

It should be emphasized that these risks mainly apply to chronic and prolonged exposure to corticosteroids. In contrast, short‐term burst therapy has shown promising results in acute HF, with the CORTAHF trial^117^ and related studies reporting improved decongestion and haemodynamic outcomes without excess safety concerns.^118^

*Clinical advice: Avoid high‐dose or prolonged glucocorticoid therapy in patients with or at risk of HF; use the lowest effective dose when unavoidable. Monitor closely when prescribing fludrocortisone, as excess can precipitate or worsen HF and antagonize mineralocorticoid receptor antagonists. Short‐term corticosteroid therapy may be considered in selected patients with acute HF under specialist supervision*.

## Anticancer medications

### Anthracyclines

Anthracyclines remain a cornerstone of chemotherapy regimens for a wide range of malignancies. However, their well‐established cardiotoxic potential poses a major challenge in oncologic and cardiologic practice.^119^ Cardiotoxicity results from a multifaceted mechanism including topoisomerase IIβ inhibition in cardiomyocytes, generation of reactive oxygen species (ROS), mitochondrial dysfunction, and the formation of cardiotoxic alcohol metabolites via carbonyl reductases.^120^ These mechanisms culminate in cellular injury, contractile dysfunction, and ultimately HF. The anthracycline class comprises both traditional agents, such as doxorubicin and daunorubicin, and more recent derivatives, including epirubicin, idarubicin, and mitoxantrone.^3^

Anthracycline‐induced cardiotoxicity (AIC) presents along a spectrum: (i) acute toxicity (hours to days) with arrhythmias and myocarditis; (ii) early‐onset chronic toxicity (within the first year); and (iii) late‐onset chronic toxicity (>1 year), often manifesting as progressive, sometimes irreversible HF.^119^

Recent longitudinal data confirm that AIC can emerge decades post‐therapy. In a contemporary cohort of >15 000 cancer survivors, the 20‐year cumulative incidence of HF exceeded 5% in those exposed to >250 mg/m^2^ doxorubicin equivalents, with risk extending into adulthood for childhood cancer survivors.^121^

Building on prior findings, a more recent pooled retrospective analysis of 630 adult patients across three clinical studies reported a higher overall incidence of AIC at 5.1%. This study reinforced the dose–response association, estimating a 5% incidence at 400 mg/m^2^, 16% at 500 mg/m^2^, and 26% at 550 mg/m^2^ of cumulative doxorubicin exposure.^122^

Dexrazoxane, a topoisomerase II inhibitor and iron chelator, has shown efficacy in reducing cardiotoxicity without compromising oncologic efficacy in large meta‐analyses (HR 0.32, 95% CI 0.21–0.49).^123^ Current ESC guidelines support its use in adult patients receiving >300 mg/m^2^ doxorubicin, especially when continued anthracycline use is necessary.^119^

A pooled analysis of five randomized controlled trials showed a 60% reduction in clinical HF compared to conventional formulations (RR 0.40, 95% CI 0.24–0.67).^124^

The STOP‐CA trial further explored pharmacologic prevention strategies by evaluating the role of atorvastatin (40 mg daily) in patients receiving anthracycline‐based chemotherapy for lymphoma. In this randomized trial, atorvastatin significantly reduced the incidence of a ≥10% decline in LVEF compared to placebo (9% vs. 22%, relative risk 0.42; *p* = 0.002), providing strong evidence for statins as a feasible cardioprotective approach in this population.^125^

Neurohormonal blockade with angiotensin‐converting enzyme inhibitors and β‐blockers (especially carvedilol) has been studied both prophylactically and therapeutically. The CECCY and OVERCOME trials demonstrated that early initiation of enalapril and carvedilol preserved LVEF and reduced clinical HF incidence.^119^, ^126^, ^127^ These findings have informed guideline recommendations for prophylactic use in high‐risk patients.

*Clinical advice: Anthracyclines carry a significant risk of dose‐dependent cardiotoxicity, manifesting acutely or years after treatment, often leading to HF. Cardioprotection strategies are essential. Close cardiac monitoring and timely intervention are essential in patients undergoing anthracycline chemotherapy*.

### Alkylating agents

Alkylating agents—particularly cyclophosphamide, ifosfamide, and mitomycin C—remain key treatments for various malignancies but are associated with acute HF, often dose‐dependent and mechanistically distinct from anthracycline‐induced cardiotoxicity.

#### Cyclophosphamide

This prodrug, activated in the liver to phosphoramide mustard, is linked to acute cardiotoxicity, especially at high doses used in conditioning regimens before haematopoietic stem cell transplantation.^128^ Cardiotoxicity results from oxidative stress, mitochondrial dysfunction, endothelial injury, and activation of the NLRP3 inflammasome. Histopathology often reveals haemorrhagic myocarditis and myocardial oedema.^129^

Heart failure occurs in 17–28% of high‐dose cases, with subclinical LVEF decline in up to 50%. Onset is typically within 1–10 days and can be fatal. Risk factors include older age, prior radiation, and cumulative dosing.^130^ Mitigation strategies include limiting doses (<120–170 mg/kg), fluid management, pre‐treatment imaging, and avoiding concomitant cardiotoxins. Dexrazoxane may offer cardioprotection, though data are limited.^131^

#### Ifosfamide

The cyclophosphamide analogue, ifosfamide is less frequently associated with HF but shares similar mechanisms. In small studies, HF occurred in 17% of patients receiving >12.5 g/m^2^. Toxicity appears oxidative stress‐mediated and typically occurs within 1–10 days, often reversible. Data remain limited, highlighting the need for prospective monitoring.^132^

#### Mitomycin C

This antitumor antibiotic induces DNA cross‐linking via ROS generation, particularly under aerobic conditions in cardiomyocytes.^133^ Endothelial dysfunction may underlie early cardiac injury, exacerbated by CV risk factors. Unlike anthracyclines, mitomycin‐related HF is often reversible. Risk increases when combined with anthracyclines, with HF observed in 9.5% (monotherapy) and 15.3% (combined therapy) of patients.^134^

*Clinical advice: Alkylating agents pose a notable but underrecognized risk for acute HF, particularly in high‐dose settings. Cyclophosphamide is the most implicated, with pathogenesis driven by oxidative and endothelial injury. Prevention hinges on dose control, risk stratification, and early cardiac monitoring. Further research is needed to elucidate molecular mechanisms and evaluate targeted cardioprotective strategies*.

### Antimetabolites

Fluoropyrimidines—especially 5‐fluorouracil and capecitabine—inhibit thymidylate synthase, disrupting DNA synthesis. Cardiotoxicity, primarily due to coronary vasospasm, is well‐documented, with reported incidence ranging from 1.2% to 18%, particularly during continuous infusions.^135^

Vasospastic angina is the most common presentation, but Takotsubo syndrome, myocarditis, and HFrEF have also been observed. Apical ballooning on imaging is typical in Takotsubo cases, with most patients recovering cardiac function within weeks of drug cessation.^136^

Subclinical myocardial dysfunction may be detected via global longitudinal strain, though prospective data are limited. Rechallenge strategies using CCBs or nitrates are based on anecdotal evidence and expert consensus, lacking support from randomized trials.^137^

*Clinical advice: 5‐fluorouracil and capecitabine can cause cardiotoxicity primarily through coronary vasospasm, leading to angina, myocarditis, Takotsubo syndrome, or HFrEF. Patients receiving continuous infusions are at higher risk. Upon symptoms or cardiac dysfunction, immediate drug cessation is essential*.

### Targeted therapies

Targeted cancer therapies, including monoclonal antibodies and tyrosine kinase inhibitors (TKIs), have significantly improved oncologic outcomes but are associated with cardiotoxicity, particularly LV dysfunction and HF.^119^

#### HER2‐targeted therapies

##### Trastuzumab

This monoclonal antibody inhibits human epidermal growth factor receptor 2 (HER2) signalling, crucial for cardiomyocyte survival.^138^ When added to anthracycline‐based regimens, it raises cardiotoxicity risk to 27%, with symptomatic HF in 16%.^139^ Meta‐analyses show a fivefold increased HF risk. Most events occur within 12 months and are reversible in ~80% of cases, though recurrence or persistent dysfunction is not uncommon.^140^ High‐risk patients include those with prior anthracycline exposure, low baseline LVEF, or comorbidities. Preventive use of neurohormonal agents shows limited efficacy but may help preserve LVEF. Emerging data suggest exercise may mitigate global longitudinal strain deterioration during therapy.^141^

##### Pertuzumab

Pertuzumab blocks HER2 dimerization and has a favourable cardiac safety profile.^142^ In trials such as CLEOPATRA and APHINITY, symptomatic HF occurred in 1% or less, mostly reversible.^143^, ^144^ Meta‐analysis confirms a slight increase in HF risk (RR ~2) but with a low absolute incidence (~0.4%).^145^ Risk remains manageable with standard monitoring.

##### Lapatinib

The dual HER1/HER2 TKI, lapatinib has low cardiotoxicity.^146^ Pooled data show symptomatic HF in 0.2% and LVEF decline in 1.4%, with reversibility in most cases.^147^ Risk is higher with prior trastuzumab or anthracycline use. Long‐term follow‐up from ALTTO confirms a low cumulative cardiac risk, supporting its use in patients with prior cardiotoxicity, given close LVEF monitoring.^148^

*Clinical advice: HER2‐targeted therapies carry varying risks of cardiotoxicity, primarily LV dysfunction and HF. Routine cardiac surveillance and individualized risk assessment guide safe use of these agents*.

#### Vascular endothelial growth factor inhibitors and multikinase tyrosine kinase inhibitors

##### Bevacizumab

The anti‐vascular endothelial growth factor (VEGF)‐A antibody bevacizumab is associated with a 1.6% incidence of HF (vs. 0.4% in controls).^149^ Most cases are reversible, but some show persistent fibrosis on cardiac magnetic resonance, likely due to microvascular ischaemia and endothelial dysfunction.

##### Sunitinib

The multitarget TKI inhibiting VEGF receptor/platelet‐derived growth factor receptor, sunitinib causes HF in up to 10% (grade ≥3 in 2%). Mechanisms include mitochondrial dysfunction, AMPK inhibition, and ferroptosis.^150^ HF risk is highest early in therapy and linked to hypertension and coronary artery disease.^151^ Pre‐clinical studies support protective roles of AMPK activators and agents like baicalin.^152^

##### Sorafenib

Sorafenib, another VEGF‐targeting TKI, is less cardiotoxic than sunitinib but still increases HF risk (OR 3.5).^153^ Mechanisms include calcium handling defects and mitochondrial injury. Severe but reversible HF cases have been reported, especially in those with underlying cardiac disease.

#### Others

Erlotinib and pazopanib also carry HF risk. Erlotinib's effects are rare and mostly reversible, while pazopanib has a moderate risk, with occasional severe events.^154^

*Clinical advice: VEGF inhibitors and multikinase TKIs carry variable risks of HF and cardiotoxicity. Routine CV assessment and proactive management of risk factors are recommended when using these agents*.

### Taxanes

Paclitaxel and docetaxel stabilize microtubules, halting cell division. Their cardiotoxicity—mainly mild and reversible—is driven by oxidative stress and mitochondrial dysfunction, leading to endothelial injury, apoptosis, and occasional arrhythmias.^155^ Cardiac dysfunction is rare but may occur when combined with anthracyclines, as paclitaxel impairs doxorubicin clearance.

A trial of docetaxel‐trastuzumab reported asymptomatic ≥15% LVEF declines in 8% of patients, mostly with prior anthracycline use.^156^ A 2025 retrospective study (*n* = 68) showed no cardiac changes in taxane‐only patients; combinations led to transient ~3% LVEF drops.^157^

A meta‐analysis of 86 randomized controlled trials (~100 000 patients) found no significant increase in clinical HF with taxanes, though paclitaxel may pose a slightly higher risk than docetaxel.^158^

*Clinical advice: Taxanes (paclitaxel and docetaxel) generally have a low risk of cardiotoxicity, with mainly mild, reversible effects. Close monitoring of LVEF is advisable in patients receiving combined therapy, especially those with prior anthracycline exposure*.

### Other anticancer agents

Several non‐anthracycline/non‐TKI drugs also cause cardiotoxicity:

- Immunomodulators (thalidomide, lenalidomide): linked to thrombosis, arrhythmias, and rare HF, especially in older or steroid‐treated patients.^159^
- Immune checkpoint inhibitors: rare but severe immune‐mediated myocarditis (~1% incidence, >40% mortality in fulminant cases).^160^
- Proteasome inhibitors (bortezomib, carfilzomib): associated with hypertension, arrhythmias, and LV dysfunction; carfilzomib carries higher HF risk, likely via oxidative and mitochondrial injury.^161^

## Conclusion

Heart failure management is increasingly challenged by the complexity of polypharmacy and the frequent use of medications that may precipitate or worsen cardiac dysfunction. This consensus document highlights a broad spectrum of commonly prescribed agents—from antidiabetic and antihypertensive drugs to antiarrhythmics, antimicrobials, neurological, psychiatric, anaesthetic, and anticancer therapies—that carry potential risks in patients with HF (*Graphical Abstract*). Awareness of these agents, their mechanisms of harm, and the clinical scenarios in which they are most dangerous is essential to prevent avoidable deterioration. Importantly, safer alternatives are often available and should be preferred whenever possible.

The integration of systematic medication reviews, collaboration among cardiologists, internists, pharmacists, and other specialists, and individualized therapeutic decisions are crucial to minimizing iatrogenic harm. A proactive approach—avoiding high‐risk drugs, closely monitoring patients when potentially harmful medications are unavoidable, and promptly recognizing adverse effects—can substantially reduce HF‐related morbidity and mortality. By improving awareness of pharmacologic pitfalls, this document seeks to support clinicians in optimizing therapy, improving safety, and ultimately enhancing outcomes for patients living with HF.
